# Road traffic injury Epidemiology

**Published:** 2019-08

**Authors:** Hamid Soori

**Affiliations:** ^*a*^ Professor of Epidemiology, Safety Promotion and Injury Prevention Research center, Shahid Beheshti University of Medical Sciences, Tehran- Iran.

## Abstract

About 25% of all fatal injuries or 1.35 million deaths on the world’s roads are due to road traffic injuries (RTIs). For every road traffic fatality, at least 20 people sustain non-fatal injuries. Deaths from RTIs has been increased from 2015 to 2018 global report. Distribution of these deaths varies by epidemiological patterns. For instance, the global road traffic fatality rate is 18 per 100 000 population, however, 20.1 in middle-income countries while 8.7 in high-income ones. About 80% of road traffic deaths occur in middle-income countries, with 72% of the world’s population, and only 52% of the world’s registered vehicles. Road traffic crashes cost countries up to 4-8% of their Gross National Product (it is 4.46% in Iran).

The patterns of RTIs vary in different countries and regions. For example, 38% of all African road traffic deaths occur among pedestrians, while 36% of road traffic deaths in the Western Pacific Region are among motorcyclists. Pedestrians are the largest group of vulnerable road users in most countries. Close to 40% of all pedestrians killed belong to the age group 65+. Inappropriate behaviour of road users, such as excessive and inappropriate speed, driving under the influence of alcohol and/or drugs and the non-use of safety equipment such as seat belts and crash helmets, remain important contributory factors in fatal crashes and for injuries. Almost 60% of RTI deaths are among 15–44 year olds.

Common Risk factors on RTIs include factors influencing; exposure to risk (e.g. rapid motorization, increased need for travel and choice of less safe forms of travel), crash involvement (e.g. Driver fatigue, speeding, inadequate visibility), injury severity (e.g. non-use of crash helmets and seatbelts), and post-crash injury outcome (pre-hospital and hospital care factors).

Deaths from RTIs are one of the top causes of deaths in Iran and are higher than the region and world rates. The pace of legislative change is too slow. Countries need to increase adoption of comprehensive legislation relating to key risk factors for road traffic injuries. There are minimum elements needed in national laws related to the key risk factors (speed, drink–driving, motorcycle helmets, seat-belts and child restraints), and these should be rolled out in all countries. In addition, experience from high-performing countries has demonstrated that a continual process of legislative review to further strengthen laws can lead to additional benefits. Education is a key component of road safety and five key interventions including control of speeding, drinking and driving, seatbelt use, helmet use and child Restraint use should be addressed to reduce a significant amount of RTIs. Crude experiences, trial and error could not be effective and cost effective for reducing RTIs. Having valid and reliable data, good domestic research and evidence-based decision making are essential approaches for preventing of fatal RTI particularly in low and middle income countries.

**Keywords:**

Road traffic, epidemiology, death, injury, risk factors

